# *NPM1* mutation reprograms leukemic transcription network via reshaping TAD topology

**DOI:** 10.1038/s41375-023-01942-9

**Published:** 2023-06-26

**Authors:** Qian Lai, Karina Hamamoto, Huacheng Luo, Zachary Zaroogian, Caixian Zhou, Julia Lesperance, Jie Zha, Yi Qiu, Olga A. Guryanova, Suming Huang, Bing Xu

**Affiliations:** 1 Department of Hematology, The First affiliated Hospital of Xiamen University, Xiamen University School of Medicine, Xiamen, 361003 China; 2 Division of Pediatric Hematology/Oncology, Pennsylvania State University College of Medicine, Hershey, PA 17033 USA; 3 Department of Pharmacology and therapeutics, University of Florida College of Medicine, Gainesville, FL 32610 USA; 4 UF Health Cancer Center, Gainesville, FL 32610 USA; 5 Department of Cellular and Molecular Physiology, Pennsylvania State University College of Medicine, Hershey, PA 17033 USA; 6 Present Address: The Cancer Hospital of the University of Chinese Academy of Sciences, Hangzhou Institute of Medicine, Hangzhou, Zhejiang 310022 China

**Keywords:** Acute myeloid leukaemia, Haematopoietic stem cells

## Abstract

C-terminal mutation of *Nucleophosmin 1* (*NPM1*^*C+*^) was thought to be a primary driving event in acute myeloid leukemia (AML) that reprograms leukemic-associated transcription programs to transform hematopoietic stem and progenitor cells (HSPCs). However, molecular mechanisms underlying *NPM1*^*C+*^-driven leukemogenesis remain elusive. Here, we report that *NPM1*^C+^ activates signature HOX genes and reprograms cell cycle regulators by altering CTCF-driven topologically associated domains (TADs). Hematopoietic-specific *NPM1*^*C+*^ knock-in alters TAD topology leading to disrupted regulation of the cell cycle as well as aberrant chromatin accessibility and homeotic gene expression, which results in myeloid differentiation block. Restoration of NPM1 within the nucleus re-establishes differentiation programs by reorganizing TADs critical for myeloid TFs and cell cycle regulators that switch the oncogenic MIZ1/MYC regulatory axis in favor of interacting with coactivator NPM1/p300, and prevents *NPM1*^*C+*^-driven leukemogenesis. In sum, our data reveal that *NPM1*^C+^ reshapes CTCF-defined TAD topology to reprogram signature leukemic transcription programs required for cell cycle progression and leukemic transformation.

## To the Editor

**Fig. 1:**
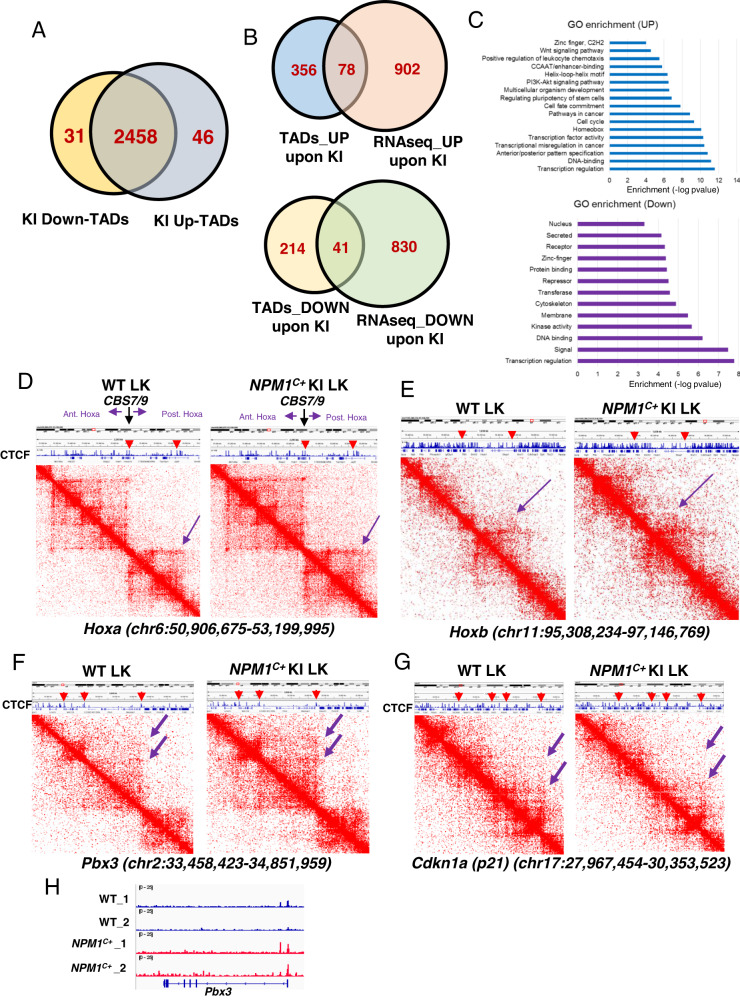
*NPM1*^*C+*^ KI alters hematopoietic TAD resulted in *NPM1*^*C+*^ signature gene expression profile. **A** Overlapping of mouse BM HS/PC TADs and *NPM1*^*C+*^ KI HS/PC TADs identified by Hi-C assay using WT and *NPM1*^*C+*^ cKI mouse BM LK cells of which NPM1^C+^ was induced at 8 weeks old animals for 4 months. **B** Overlapping of enhanced or reduced TAD encompassed gene and upregulated or downregulated genes by RNA-seq upon *NPM1*^*C+*^ KI in BM HS/PCs, respectively. **C** GO term analysis of overlapped enhanced or reduced TAD encompassed gene and upregulated or downregulated genes by RNA-seq upon *NPM1*^*C+*^ KI in BM HS/PCs, respectively. NPM1^C+^ was induced at 8 weeks old animals for 4 months. **D** Hi-C interacting maps in part of mouse chromosome 6 regions containing *Hoxa* locus comparing WT and *NPM1*^*C+*^ KI mouse BM HS/PCs. Publicly available CTCF ChIP-seq data (GSM918744) in MEL cells was integrated on top with Hi-C data. Note: CTCF binding at the boundaries of altered TADs or sub-TADs (red arrow heads). **E** Hi-C interacting maps in part of mouse chromosome 11 regions containing *Hoxb* locus comparing WT and *NPM1*^*C+*^ KI mouse BM HS/PCs. Publicly available CTCF ChIP-seq data (GSM918744) in MEL cells was integrated on top with Hi-C data. Note: CTCF binding at the boundaries of altered TADs or sub-TADs (red arrow heads). **F** Hi-C interaction map at the *Pbx3* locus comparing WT and *NPM1*^*C+*^ KI in BM HS/PCs. Publicly available CTCF ChIP-seq data (GSM918744) in MEL cells was integrated on top with Hi-C data. Note: CTCF binding at the boundaries of altered TADs or sub-TADs (red arrow heads). **G** Hi-C interaction map at the *Cdkn1a* locus comparing WT and *NPM1*^*C+*^ KI in BM HS/PCs. Publicly available CTCF ChIP-seq data (GSM918744) in MEL cells was integrated on top with Hi-C data. Note: CTCF binding at the boundaries of altered TADs or sub-TADs (red arrow heads). **H** ATAC-seq chromatin accessibility of *Pbx3* gene locus compared WT and *NPM1*^*C+*^ KI in BM HS/PCs.

Mutation of *NPM1* results in cytoplasmic sequestration of NPM1^C+^ protein that maintains a unique expression pattern of *HOXA/B* genes and *MEIS1* oncogene by directly binding to chromatin target sites to facilitate AML leukemogenesis [1–3]. However, molecular mechanisms underlying *NPM1*^*C+*^-driven gene regulatory networks and leukemogenesis remain elusive. Since wild-type (WT) NPM1/NPM1^C+^-associates with CTCF and may influence CTCF-mediated loops by anchoring for CTCF cluster [4, 5], it has been long speculated that NPM1^C+^ mislocalization may alter CTCF-mediated gene expression. We test whether mislocalization of NPM1^C+^ to cytoplasm alters CTCF-mediated three-dimensional (3D) topologically associated domains (TADs) by Hi-C comparing mouse bone marrow (BM) Lin^-^cKit^+^ (LK) stem- and progenitor-enriched cells purified from WT and *NPM1*^*C+*^-KI animals induced for NPM1^C+^ expression for 4-months (Table S1 listed kits, constructs, cells, antibodies). Mice conditionally expressing *NPM1*^*C+*^ in the BM develop a myeloproliferative neoplasm/myelodysplasia-like condition with a long latency characterized by enlarged spleens, leukocytosis, and thrombocytopenia [6, 7]. We confirmed that *NPM1*^*C+*^ cKI aberrantly activated *Hoxa*/*Hoxb* and *Meis1* genes that coincided with a gain of chromatin accessibility as seen by RNA-seq and ATAC-seq (Fig. S1A, B) [1, 8]. NPM1^C+^ expression led to an increase of 46 TADs and a decrease of 31 TADs (Fig. 1A). The increased TADs encompassed 434 genes of which approximately 18% (78 genes) overlapped with genes increased in expression upon *NPM1*^*C+*^-cKI measured by RNA-seq (Fig. 1B, top). Theses overlapping genes are mainly NPM1^C+^-signature genes involved in pathways regulating transcription, anterior/posterior patterning, homeobox genes, and cell cycle (Fig. 1C, top). Top TADs strengthened by the *NPM1*^*C+*^*-*cKI included *Hoxa/b*, and *Pbx3* loci (Fig. 1D–F). Many of these genes are specifically involved in unique NPM1^C+^-driven leukemic transcription program [1, 8]. In contrast, the decreased TADs included cell cycle kinase inhibitors (CDKIs), such as *Cdkn1a* (p21) gene (Fig. 1G). The altered TADs or sub-TADs were defined by CTCF binding when we integrated with public available mouse CTCF ChIP-seq dataset (GSM918744), whereby CTCF bound to the altered TAD or sub-TAD boundaries (Fig. 1D-G, Red arrowheads). As expected, chromatin accessibility in *Hoxa/b* and *Pbx3* loci were coordinately increased (Figs. S1B, 1H). In light of NPM1^C+^ directly interacting with CTCF and binding to its chromatin targets [2, 3, 5], NPM1^C+^ and CTCF may coordinate to remodels NPM1^C+^-signature gene TADs resulting in altered chromatin accessibility and oncogenic gene expression signatures.

**Fig. 2:**
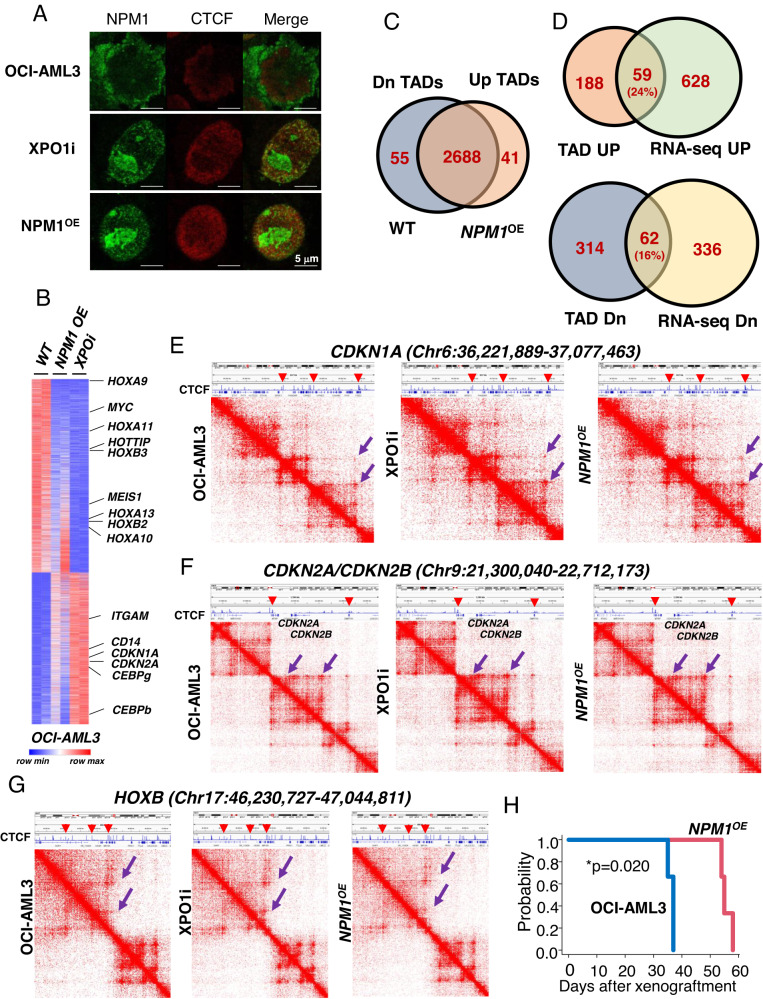
Nuclear retention of NPM1 enhanced NPM1 and CTCF interaction and alters leukemic TAD formation and gene expression. **A** Immunofluorescence staining of NPM1 and CTCF localization and association upon XPO1i treatment or WT NPM1 expression. Note: The NPM1 antibody used recognized both WT and NPM1^C+^ mutant. **B** Heat map of RNA-seq analysis shows upregulated and downregulated genes upon WT NPM1 overexpression or XPO1i treatment in OCI-AML3 cells. **C** Overlapping of TADs identified from WT OCI-AML3 cells and *NPM1*^*OE*^ OCI-AML3 cells by Hi-C assay. **D** Overlapping of enhanced or reduced TAD encompassed gene and upregulated or downregulated genes by RNA-seq upon *NPM1*^*OE*^ in OCI-AML3 cells. **E** Hi-C interacting maps in part of human chromosome 6 regions containing *CDKN1A* gene comparing WT, XPO1i treated, or *NPM1*^*OE*^ OCI-AML3 cells. Publicly available CTCF ChIP-seq data in human K562 cells was integrated on top with Hi-C data (GSM1010820). Note: CTCF binding at the boundaries of altered TADs or sub-TADs (red arrow heads). **F** Hi-C interacting maps in part of human chromosome 9 regions containing *CDKN2A/CDNK2B* genes comparing WT, XPO1i treated, or *NPM1*^*OE*^ OCI-AML3 cells. **G** Hi-C interaction map at the *HOXB* loci comparing WT, XPO1i treated, or *NPM1*^*OE*^ OCI-AML3 cells. Publicly available CTCF ChIP-seq data in human K562 cells was integrated on top with Hi-C data (GSM1010820). Note: CTCF binding at the boundaries of altered TADs or sub-TADs (red arrow heads). **H** Survival of NSG mice (*n* = 5) transplanted with WT or *NPM1*^*OE*^ OCI-AML3 cells. Data are presented as mean ± SD. *p ≤ 0.05; **p ≤ 0.01; ***p ≤ 0.001.

We next transfected OCI-AML3 cells with vector caring WT *NPM1* cDNA to restore nuclear NPM1 (*NPM1*^*OE*^), or treated OCI-AML3 cells with 50 nM XPO1 inhibitor (XPO1i), selinexor, for 3 days to block the NPM1^C+^ cytoplasmic export, and test if restoration of nuclear NPM1 reversed *NPM1*^*C+*^*-*associated HOX expression signature and TAD formation. Immunofluorescent staining confirmed that XPO1i or *NPM1*^*OE*^ resulted in nuclear localization of NPM1 and enhanced its colocalization with CTCF (Fig. 2A). Nuclear retention of NPM1 reduced *MYC*, *MEIS1*, and *HOX* gene expression, while increased CDKIs, *CDKN1A* and *CDKN2A* genes (Fig. 2B), consistent with the transcriptomic GO analysis (Fig. S2A, B).

Next, we carried out Hi-C and RNA-seq experiments in WT, XPO1i-treated or *NPM1*^*OE*^ OCI-AML3 cells, and integrated our Hi-C data with available CTCF ChIP-seq profiles generated in K562 cells (GSM1010820). *NPM1*^*OE*^ altered 96 TADs defined by CTCF boundaries. Among those, 55 TADs were decreased, while 41 TADs were increased by *NPM1*^*OE*^ (Fig. 2C). Similarly, XPO1i treatment altered 97 CTCF-defined TADs (59 decreased TADs and 38 increased TADs, data not shown). We further integrated the transcriptomic data with Hi-C datasets from XPO1i-treated or *NPM1*^*OE*^ OCI-AML3 cells. Upon *NPM1*^*OE*^, 24% of genes residing within up-regulated TADs were transcriptionally up-regulated determined by RNA-seq (Fig. 2D, top), and they were predominantly involved in cell cycle, cell differentiation, apoptosis, DNA damage and repair, and transcription regulation (Fig. S2C, left). Only 2.4% of genes residing within up-regulated TADs were transcriptionally down-regulated (Fig. S2D, Left). On the other hand, 16% of genes encompassed within reduced TADs were significantly down-regulated according to their expression levels (Fig. 2D, bottom), and were enriched in pathways involved in HOX, pathways in cancer, PI3K-AKT signaling, Wnt signaling, positive regulation of ERK1/ERK2, transcriptional regulation, cell cycle, and positive regulation of cell proliferation (Fig. S2C, right). Only 4.5% of genes residing within down-regulated TADs were transcriptionally up-regulated (Fig. S2D, Right). XPO1i treatment reveal very similar TADs and transcriptomic changes (Fig. S2E, F). Thus, NPM1^C+^-driven TAD alteration may be critical for the NPM1^C+^ signature gene expression.

In particular, Hi-C data revealed that top ten upregulated TADs encompassed genes encoding CDKIs, *p21*, *p15*, *p16*, and *p27*, while top ten reduced TADs included HOXB in both XPO1i or *NPM1*^*OE*^ (Fig. 2E–G). These altered TADs or sub-TADs are defined by CTCF binding when we integrated with CTCF ChIP-seq data from K562 cells (GSM1010820), whereby CTCF bound to boundaries of the altered TADs or sub-TADs (Fig. 2E–G; Red arrowheads). Interestingly, CDKI genes are regulated by MYC-MIZ-1 transcription axis that is involved in control of LSC proliferation and cell cycle progression [9].

To further support that NPM1 nuclear relocalization is in part inducing genome TAD reorganization, we performed time course experiments by treated OCI-AML3 cells with XPO1i for 0, 1, 3, 6, 24, and 48 h. We observed a significantly increase in nuclear localization of NPM1^C+^ after 24 h treatment by WB (Fig. S3A) and immunofluorescent-staining (Fig. S3B, C). Time course 3 C assay revealed that CTCF-loops encompassed anterior HOXB genes (*HOXB1*-*HOXB5*) were significantly impaired as early as 3 hr upon blocking NPM1^C+^ cytoplasmic localization, while the CTCF mediated looped interactions encompassing *CEBPA/G* genes were increased immediately upon blockage of NPM1^C+^ cytoplasmic translocation (Fig. S3D, Table S2 listed all primers/probes). Coordinately, expression levels of CDKIs and *CEBPA* genes were only upregulated upon 24 hr and 3 days XPO1i treatment, respectively (Fig. S3E), suggesting that TAD reorganization precede gene expression changes.

It has been proposed that MIZ-1 activates its target genes including *CDKN1A* and *CDKN2B* by associating with NPM1 and p300, while in MYC transformed cancer cells, association of MYC and MIZ-1 repels NPM1 and p300 leading to aberrant proliferation [10, 11]. We further tested whether restoration of nuclear NPM1 switches the MIZ-1 transcription circuit from repression to activation of CDKIs by disassociating from MYC in favor of interacting with NPM1. Although MIZ-1 level remains relatively consistent upon XPO1i treatment or *NPM1*^*OE*^, these interventions promoted MIZ-1 interacting with NPM1 in nucleus compared to untreated OCI-AML3 cells (Fig. S4A, bottom). As a consequence, the amount of MIZ-1-associated p300 was increased, while MIZ-1 binding to MYC and G9a, a MYC-associated H3K9-metyltransferase required for repression, was markedly decreased upon nuclear retention of NPM1 (Fig. S4A). RT-qPCR and RNA-seq revealed that expression of several MIZ-1 targets, *CDKI* and *CEBP* genes, was significantly increased (Fig. 2B, S3E, S4B). ChIP-qPCR further revealed that MYC and G9a recruitment to these loci was significantly reduced comparing to the constant MIZ-1 chromatin binding (Fig. S4C–E), while recruitment of NPM1 and p300 was significantly increased upon XPO1i treatment or *NPM1*^*OE*^ (Fig. S4F, G). As a consequence, active H3K27ac chromatin marks were increased (Fig. S4H) while H3K9me2 repressive chromatin marks were significantly reduced at these MIZ-1 target genes (Fig. S4I) comparing to control IgG (Fig. S4J), However, we cannot distinguish whether WT-NPM1 or NPM1^C+^ regulates *CDKI* and *CEBP* genes upon nuclear retention of NPM1 because the antibody used recognized both forms.

Although NPM1 antibody that we used cannot distinguish WT from mutant protein and their role in CDKI and CEBP gene regulation, analyzing Bru-seq data of WT and XPO1i treated OCI-AML3 cells from recent published data [2] also revealed that the nascent transcripts of *CDKN1A* and *CEBPA* genes are consistently enhanced by XPO1i treatment (Fig. S4K), supporting that nuclear restoration of NPM1 reverses aberrant TAD topology and switches the MYC-MIZ-1 axis to restore cell cycle control and myeloid differentiation. However, it remains to be determined how NPM1^c+^/NPM1 were targeted to distinct chromatin sites to differentially activate oncogenic vs myeloid differentiation pathway. Given that NPM1 interacts with MIZ-1 and chromatin bound CRM1/XPO1 recruits NPM1^C+^ onto *HOX* cluster [2, 3, 10, 12]. It is reasonable to suspect that MIZ-1 or CRM1/XPO1 directs NPM1 or NPM1^C+^ to the intended chromatin targets, respectively. In addition, HOX associated lncRNAs, *HOTTIP* and *HOXBLINC*, are aberrantly activated in NPM1^C+^-AML cells that may also play a role in NPM1^C+^-driven oncogenic HOX gene expression [8, 13, 14].

Consistent with upregulation of CDKIs along with myeloid TFs, CEBPA and CEBPD, nuclear retention of NPM1 promoted OCI-AML3 cell differentiation seen by increased expression of myeloid-specific surface markers CD11b and CD14 (Fig. S5A–C). Furthermore, nuclear retention of NPM1 consistently increased the proportion of cells in G1 phase at the expense of cell in the S phase and G2/M phase transition (Fig. S5D), which coincided with enhanced apoptosis (Fig. S5E) and decreased cellular proliferation (Fig. S5F). Of note, XPO1i impairs OCI-AML3 cell growth and differentiation in a p53 independent manner [15]. We further test whether restored NPM1 in nuclei of *NPM1*^*C+*^ AML cells affects leukemogenesis and survival of AML mouse models in vivo, we transplanted 1 × 10^6^ WT, *NPM1*^*OE*^ OCI-AML3 cells into NSG mice. All animals transplanted with WT OCI-AML3 cells died around 38 days after transplantation, while mice that received OCI-AML3 cells with *NPM1*^*OE*^ survived between 54–58 days (Fig. 2H). Thus, nuclear NPM1 localization mitigates leukemogenesis in a xenograft mouse model of AML with *NPM1*^*C+*^, perhaps blocking NPM1^C+^-driven TAD topology and signature gene expression and restoring cell cycle control and myeloid differentiation.

## Supplementary information

Supplementary Information-NPM1

## Data Availability

Sequence reads have been deposited in the National Center for Biotechnology Information GENE expression Omnibus (NCBI GEO) under accession number GSE208022.
