# Supplementary material for: NPM1 mutation reprograms leukemic transcription network via reshaping TAD topology
Source: Leukemia. 2023 Jun 26;37(8):1732–6. doi: 10.1038/s41375-023-01942-9 (PMC10400418; doi:10.1038/s41375-023-01942-9)
Supplement: Supplementary file 1 — Supplementary Information-NPM1 [file 41375_2023_1942_MOESM1_ESM.pdf]

NPM1 mutation reprograms leukemic transcription network via reshaping TAD topology

Qian Lai[1,2,#], Karina Hamamoto[2,#], Huacheng Luo[2,#], Zachary Zaroogian[3], Caixian Zhou[2], Julia Lesperance[2], Jie Zha[1], Yi Qiu[4], Olga A Guryanova[3], Suming Huang[2*], and Bing Xu[1,*]

Supplementary Tables:

Supplementary Table 1. List of antibodies and reagents

Resource	Catalog#	Source
**Regents**		
Quick-RNA MiniPrep	R1054	ZYMO research
High Capacity RNA-to-cDNA Kit	4387406	Thermo Fisher
SsoAdvanced Universal SYBR® Green	1725274	BioRad
Illumina TruSeq mRNA sample preparation kit	20020594	Illumina
IDT for Illumina TrueSeq DNA UD Indexes	20023784	Illumina
Qubit™ dsDNA HS Assay Kits	Q32854	Invitrogen
AMPure XP Beads	A63881	Beckman Coulter
MEM alpha (500ml x 6)	SH3026501	HyClone products (Cytiva)
Fetal Bovine Serum (Heat Inactivated)	S1620	Biowest
Penicillin-Streptomycin (10,000 U/mL)	15140148	Thermo Scientific
Formaldehyde	F79-500	Fisher Scientific
Arima HiC+ kit	A410030	Arima Genomics
KAPA Hyper Prep Kit	KK8504	Kapa Biosystems
Paraformaldehyde solution 4% in PBS	sc-281692	ChemCruz
Tween 20 (100 mL)	BP337100	Fisher Scientific
Bovine Serum Albumin (BSA)	BP9703100	Fisher Scientific
Signal Immunoreaction Enhancer Solution	TYB-NKB-101T	Cosmo Bio
PBS, Phosphate Buffered Saline, 10X Solution	BP399-1	Fisher Scientific
ProLong Diamond Antifade Mountant with DAPI	P36971	Invitrogen
May Grunwald stain solution	MG500	Sigma-Aldrich
Giemsa stain solution	GS500	Sigma-Aldrich
VectaMount® Permanent Mounting Medium	H-5000-60	Vector Laboratories
Hbss, calcium, magnesium, no phenol red	14-025-076	Fisher Scientific
FxCycleTM PI/RNase Staining Solution	F10797	Invitrogen
AnnexinV, APC Ready Flow Regent	R37176	Invitrogen
BD Bovine Serum Albumin (BSA) Stain Buffer	554657	BD Pharmingen
Selinexor (KPT-300)	S7252	Selleck Chemicals
NOD.Cg-Prkdcscid Il2rgtm1Wjl/SzJ mouse	5557	the Jackson Laboratory
**Antibodies**		

NPM1(B23,N-terminal)	HPA011384	Sigma-Aldrich
Miz-1	sc-22837	Santa Cruz
G9a	ab185050	abcam
c-Myc	ab9132	abcam
P300	54062	Cell Signaling Technology
H3K27ac	ab4729	abcam
H3K9me2	ab1220	abcam
B23 (0412)	sc-47725	Santa Cruz
CTCF Antibody	2899S	Cell Signaling Technology
Anti-Ki67 antibody	ab15580	abcam
Goat anti-Rabbit IgG (H+L), Alexa Fluor 568	A-11011	Thermo Scientific
Goat anti-Mouse IgG (H+L), Alexa Fluor 488	A-11001	Thermo Scientific
Anti-alpha Tubulin antibody [DM1A]	ab7291	abcam
FITC anti-mouse/human CD11b Antibody	101205	BioLegend
APC anti-human CD14 [HCD14]	325608	BioLegend
**Plasmids**		
pEGFP-NPM WT	17578	Addgene
pEGFP-C2	V012023	NovoPro BioScience Inc.
**Cells**		
OCI-AML3	ACC-582	DSMZ

Supplementary Table 2. List of primers and probes

**primers**	**sequence**	**method**
Human P15-F	CGTTAAGTTTACGGCCAACG	RT-qPCR
Human P15-R	CCATCATCATGACCTGGATCG	RT-qPCR
Human P16-F	ATGGAGCCTTCGGCTGACT	RT-qPCR
Human P16-R	CACCAGCGTGTCCAGGAAG	RT-qPCR
Human P21-F	TCTTGTACCCTTGTGCCTCG	RT-qPCR
Human P21-R	AGAAGATCAGCCGGCGTTTG	RT-qPCR
Human CEBPA-F	AGCCTTGTTTGTACTGTATG	RT-qPCR
Human CEBPA-R	AAAATGGTGGTTTAGCAGAG	RT-qPCR
Human CEBPD-F	AGCGCAACAACATCGCCGTG	RT-qPCR
Human CEBPD-R	GTCGGGTCTGAGGTATGGGTC	RT-qPCR
Human P15 Pro-F	CCGTCGTCCTTCTGCGGCTTG	ChIP-qPCR
Human P15 Pro-R	AGTGAGGACTCCGCGACGCGT	ChIP-qPCR
Human P16 Pro-F	TCGCCAGGAGGAGGTCTGTGATTAC	ChIP-qPCR
Human P16 Pro-R	CAGGTGGGTAGAGGGTCTGCAGC	ChIP-qPCR
Human P21 Pro-F	CCGAAGTCAGTTCCTTGTGG	ChIP-qPCR
Human P21 Pro-R	CGCTCTCTCACCTCCTCTGA	ChIP-qPCR
Human CEBPA Pro-F	CCTGCCGGGTATAAAAGCTG	ChIP-qPCR

Human CEBPA Pro-R	GACTCCATGGGGGAGTTAGAG	ChIP-qPCR
Human CEBPD Pro-F	TCCGTAGACCTGGAAGAAAC	ChIP-qPCR
Human CEBPD Pro-R	CAAAAGCAGAGCAGATGGG	ChIP-qPCR
Human GAPDH Pro-F	CCACATCGCTCAGACACCAT	ChIP-qPCR
Human GAPDH Pro-R	CCCGCAAGGCTCGTAGAC	ChIP-qPCR
HOXB-B1-3C-R	GGCAGGCCTGTTTTCAAGAA	3C-PCR
HOXB-B1-3C-nest-R	TCTCCCAAGCCTCCATTTGT	3C-PCR
HOXB-B2-3C-F	GGCAAAGGGGAAATGGTGTC	3C-PCR
HOXB-B2-3C-nest-F	AAGTGGGGCTCGGTTGAC	3C-PCR
HOXB-B3-3C-R	TGCGGAAAGTGGTAAGGGAT	3C-PCR
HOXB-B3-3C-nest-R	ACATCTGTTTGCATACATTGTGT	3C-PCR
CEBPA-C1-3C-R	CCTGTGCTGAAACCCAAAAGA	3C-PCR
CEBPA-C1-3C-nest-R	GCCTGCCTTCCATTCGAATT	3C-PCR
CEBPA-C2-3C-F	AGTCTTGGTCTTGAGCTGCT	3C-PCR
CEBPA-C2-3C-nest-F	TCTTTCAAAGCCAGAACCAGG	3C-PCR
CEBPA-C3-3C-R	CACTGGGCAGAACACATCAG	3C-PCR
CEBPA-C3-3C-nest-R	CACAGACAGACGGGCTTTG	3C-PCR
DPNII-3C-digestion-test-F	TCTTTCAAAGCCAGAACCAGG	3C-PCR
DPNII-3C-digestion-test-R	TACCTCTGCGCGGAATCAC	3C-PCR
DPNII-3C-loading-ctrl-F	ATGTAGGCGCTGATGTCGAT	3C-PCR
DPNII-3C-loading-ctrl-R	TCGGCCGACTTCTACGAG	3C-PCR

Supplementary Materials and Methods:

NPM1^{C+}-expressing mouse model

Generation of a mouse line that inducibly expresses a human *NPM1*$^{C+}$ transgene has been described [1]. To achieve hematopoietic-specific expression, *NPM1*$^{C+}$ animals were intercrossed with the *Mx1*-driven Cre deletor line. Activation of the Cre-mediated excision of the Lox-Stop-Lox cassette preceding the *NPM1*$^{C+}$ transgene of 8-weeks old mice was achieved by 5 intraperitoneal injections of poly(I:C) every other day. Bone marrow cells were harvested at 6 months (after 4 months induction of *NPM1*$^{C+}$) from femurs, tibiae, and ilea of fully excised mice and littermate controls, and hematopoietic stem/progenitor populations were isolated by a two-step procedure consisting of mature hematopoietic lineage-depletion (Cat. # 19856, StemCell Technologies)

followed by cKit/CD117-enrichment (Cat. # 18757, StemCell Technologies) according to manufacturer's protocols. Efficiency of HSPC enrichment was confirmed by flow cytometry, routinely achieving >95% $cKit^+$ cells. Double-enriched *NPM1*$^{C+}$ and WT control HSPCs were then subjected to gene expression (RNA-seq) and chromatin confirmation capture (Hi-C) analyses.

Cell culture and transfection

OCI-AML3 cells were maintained in alpha-MEM (with ribo- and deoxyribonucleosides) supplemented with 20% heat inactivated fetal bovine serum and 100 U/ml of penicillin/100 μg/ml of streptomycin. For expression of *GFP-NPM1*, the plasmid *pEGFP-NPM*1 (Plasmid #17578) was purchased from Addgene (Table S1). One μg of Empty vector (pEGFP-C2) and *pEGFP-NPM*1 plasmids were transfected into OCI-AML3 cells using the Nucleofection protocol according to the manufacturer's instructions. Transfected OCI-AML3 cells were immediately transferred to 2ml of MEM alpha medium in 6 well-plate and incubated 3 hours in CO_2 incubator. After changing fresh media, the cells were cultured for 2 days. GFP positive cells were sorted by BD FACS Aria SORP high-performance cell sorter (BD) and used for experiments.

XPO1 inhibitor treatment

Selinexor (KPT-330, XPO1 inhibitor) was purchased from Selleck Chemicals (Houston, TX, US). Selinexor treatment of OCI-AML3 cells was performed with a final concentration of 50 nM for 3 days or for indicated times according to a previous study [2].

Immunofluorescence staining

The cells were resuspended in 1% BSA and centrifuged at 700 rpm for 4 min using Cytospin 4 (Thermo Scientific™). The slides were fixed with 4% PFA for 15 min and permeabilized with 0.1% Tween for 15min. After blocking in 1% BSA, the slides were incubated with primary antibody (anti-NPM1, sc47725, Santa-Cruz; anti-CTCF, 2899S, Cell Signaling Technology; Ki67, ab15580, abcam; with 1:100 dilution) at 4 degree for overnight. The slides were washed in PBS and

incubated with Alexa Fluora™488 goat anti-mouse IgG or Alexa Fluora™568 goat anti-rabbit IgG (Invitrogen). After washing in PBS, the slides were mounted using ProLong™ Diamond Antifade Mountant with DAPI (Invitrogen). For imaging, we used Leica SP8 STED 3X Inverted Confocal. Information of antibodies is listed in Table S1.

Cell cycle analysis

WT and OCI-AML3 cells treated with or without XPO1 were harvested and washed with phosphate buffered saline (PBS). The washed cells were fixed by adding 70% ethanol drop wise to the pellet with vortexing and incubated overnight at 4 °C. After fixation, cells were washed with PBS twice, treated with the staining buffer (RNase A, Triton X-100, propidium iodide) and then incubated at 37 °C for 30 min in the dark. Stained samples were analyzed on the BD Accuri™ C6 Plus flow cytometer (BD Biosciences), and cell cycle data analysis was performed using FlowJo software. Triplicate experiments were performed for each sample.

Co-immunoprecipitation (co-IP) Assay

For co-IP assays, cells were harvested by centrifugation and then washed by 1×PBS. Nuclear and cytoplasmic extracts were prepared using NE-PER™ Nuclear and Cytoplasmic Extraction Reagent kit (Thermo Scientific™). Nuclear extracts were pre-cleared by rotating with Protein A/G agarose beads (Santa Cruz, #sc-2003) for 30 min at 4°C. After centrifugation, the supernatant was transferred into two tubes and diluted to 1 ml by RIPA buffer (Thermo Scientific™). Precleared nuclear extracts were then incubated with primary antibody (anti-MIZ1 Santa Cruz #sc-22837) and normal IgG control by rotating at 4°C overnight. Protein-antibody complexes were captured by Protein A/G Agarose beads at 4°C under gentle rotation for 4 hrs. After washing the beads four times with RIPA buffer (supplemented with protease inhibitor cocktail), the associated proteins were eluted by adding 2 × SDS loading buffer and boiling for 5 min. Supernatant containing associated proteins were then subjected to Western blot analysis. Information of antibodies is listed in Table S1.

RNA isolation, quantitative RT-PCR, and RNA-sequencing

Total RNA was extracted from untreated, XPOi-treated OCI-AML3 cells and NPM1-WT overexpressing OCI-AML3 cells, and WT and *NPM1*$^{c+}$ knock-in mouse lineage-depleted cKit-enriched bone marrow (BM) cells using the RNeasy Mini kit (Qiagen) according to the manufacturer's instructions. cDNA was generated from 1 μg DNase-treated total RNA with the Superscript II Reverse Transcriptase kit (Invitrogen), and used as template for quantitative PCR.

RNA libraries were prepared according to the Illumina TruSeq mRNA sample preparation kit (Illumina, Cat# 20020594) for next generation sequencing. Firstly, polyadenylated mRNA was enriched using poly-T oligo beads, then purified mRNA was fragmented following manufacturer's instructions. Subsequently, cDNA synthesis and amplification of indexed libraries were performed. Library quality was then assessed by Qubit and Agilent Bioanalyzer. Libraries were subjected to paired-end sequencing at a 50 bp length on an Illumina NovaSeq 6000. Sequenced reads were trimmed and quality filtered using cutadapt (http://cutadapt.readthedocs.io, version 1.2.0) program [3] and aligned to the human (hg19) or mouse (mm9) genome using TopHat (version 2.0) and Bowtie2 [4-6]. FPKM (paired-end fragments per kilobase of exon model per million mapped reads) values were calculated using Cufflinks v2.2.1 and differential expression analysis was performed using Cuffdiff [7]. The heatmap was generated using cluster3.0 and treeview based on $\log_2$ transformation of the FPKM values [8]. Gene set enrichment analysis (GSEA) was performed with gene sets obtained from the Molecular Signatures Database [9]. GO analysis of differentially expressed (greater than 2 fold) genes was generated using Database for Annotation, Visualization and Integrated Discovery (DAVID) Bioinformatics Resources [10]. Sequence reads have been deposited in the NCBI GEO under accession number (GEO: GSE208022).

Chromatin immunoprecipitation (ChIP) assay

ChIP assay was performed as described previously [11]. Briefly, cells were harvested by centrifugation and then washed by 1×PBS. Cells were cross-linked by adding formaldehyde

dropwise to a final concentration of 1%. Lysed cell solution was subjected to chromatin shearing using bioruptor (Diagenode) with a 30s on and 30s off condition. Sonicated and purified chromatin from indicated cells was incubated at 4°C overnight under gentle agitation with indicated primary antibodies and 30 ul 50% pre-washed protein A/G agarose beads slurry. Bound chromatin and beads underwent buffer washing, reverse crosslinking, and DNA precipitation to elute the ChIP DNA. Immunoprecipitated DNA was then analyzed by qPCR to quantify relative enrichment of the target loci. Information of antibodies and primers are listed in Tables S1 and S2.

Assay for Transposase-Accessible Chromatin using sequencing (ATAC-seq) assay

ATAC-seq assay was performed using the Nextera DNA library preparation kit as described previously [12]. Briefly, 5×10^4 indicated cells were harvested and washed with cold PBS twice. Cell pellets were then resuspended in 50ul cold lysis buffer containing 10 mM Tris-HCl (pH 7.4), 10 mM NaCl, 3 mM $MgCl_2$, and 0.1% NP-40. Cells were spun down immediately at 500g for 10min at 4°C, and cell pellets were resuspended and treated with a transposase reaction mixture containing 2.5ul Tn5 transposase at 37°C for 30 min. The fragmented DNA was purified using the MinElute Kit (QIAGEN) and fragments were simultaneously amplified and indexed in a PCR reaction with 2x NEBNext High-Fidelity PCR master Mix (NEB). Following purification with AMPure XP beads (Beckman Coulter), libraries were quantified using qPCR Kapa Library Quantification Kit for Illumina (Roche). Quality control was done using Qubit and Agilent Bioanalyzer and libraries were subjected to 100 bp paired-end sequencing on an Illumina NovaSeq 6000.

ATAC-seq analysis

Sequenced reads were trimmed and filtered using cutadapt (http://cutadapt.readthedocs.io, version 1.2.0) [3], PCR duplicates were removed using Picard MarkDuplicates (version 2.0.1), mitochondrial reads were removed with samtools [13], and sequences underwent quality control by FastQC [14]. Reads and were mapped to the human (hg19) or mouse (mm9) reference genome

using Bowtie2 with default parameters [5]. ENCODE blacklist regions were filtered (https://sites.google.com/site/anshulkundaje/projects/blacklists). SAM files were converted to BAM files and sorted using Samtools [15]. Peak calling was performed using MACS2 with parameters (-g mm -p 1e-9 –nolambda -f BAMPE –nomodel –shiftsize 100 --extsize 200)[16]. A bigWig file, including fragment or read coverages for control and experimental datasets, was generated with the bedGraphToBigWig program, (https://www.encodeproject.org/software/bedgraphtobigwig/). Sequencing tracks were viewed using the Integrated Genomic Viewer [17]. Peak annotation was performed using HOMER [18]. DEseq2 (Benjamini-Hochberg adjusted $p < 0.05$; FoldChange≥2) was used to identify differentially accessible sites [19]. All genomics datasets were deposited in the NCBI GEO under accession number (GEO: GSE208022).

Chromosome conformation capture (3C) assay

3C assay was performed as previously described[11,20,21]. Briefly, $5x10^6$ cells were crosslinked in 2% (vol/vol) formaldehyde for 10 min at room temperature, quenched by 125 mM glycine for 5 min at room temperature, and washed with ice-cold 1x PBS twice. Cell pellets were lysed with ice-cold lysis buffer (10mM Tris pH8.0, 10mM NaCl, 0.2% Igepal CA-630, 1x Protease inhibitor) for 20min on ice, then suspended in DpnII enzymatic digestion buffer containing 0.3% SDS and incubated at 37 °C for 1 hour with shaking. The SDS was sequestered by addition of 2% Triton X-100 incubation for 1 hour at 37°C with shaking. Cell pellets were digested with 1500 U of DpnII (NEB) at 37°C overnight, and a part of undigested DNA was set up as Control 1. The enzymatic reaction was stopped for 20 minutes at 65°C, and a part of unligated DNA was set up as Control 2. Subsequently, digested DNAs were ligated with 240 U of T4 DNA ligase (Thermo Fisher) at 16°C for 16-18 hrs. Crosslink of cell pellet was reversed by addition of 3U Proteinase K (Invitrogen) and incubated at 65°C for 4 hrs, and then DNA was extracted using phenol:chloroform, precipitated with sodium acetate and glycoblue and ice-cold absolute ethanol,

and then dissolved in ddH_2O. Finally, the 3C-ligated DNA was analyzed with 3C-PCR. Information of primers is listed in Table S2.

High throughput Chromosome Conformation Capture (Hi-C) Assay

Hi-C assay was performed as described previously with Arima-HiC Kit (Cat: A410030) (https://arimagenomics.com/). Briefly, 5×10^6 indicated cells, including untreatment, XPOi treated OCI-AML3 cells and NPM1-WT overexpressing OCI-AML3 cells, and Lin^-cKit^+ BM cells derived from WT and *NPM1*$^{c+}$ knock-in mice were washed with PBS twice, crosslinked in 1% formaldehyde for 10 min at room temperature, quenched by 125 mM glycine for 5 min at room temperature, and washed with ice-cold 1x PBS twice. Cells were then resuspended in lysis buffer and incubated at 4 °C for 15 min, conditioning solution was added at 62 °C for 10 min, and the reaction was stopped by stop solution for 15 min at 37 °C. Cell pellets were then digested with the enzymatic digestion reaction buffer and restriction enzyme cocktails (Arima-HiC Kit) overnight at 37 °C with rotation and DNA was purified using AMPure XP Beads. Next, 750 ng of DNA was sheared by sonication (Bioruptor UCD200, 10 cycles 30s ON,30s OFF for 9-10 cycles) and size-selected to 200-600 bp. Sequencing libraries were then prepared from 250ng of DNA using KAPA Hyper Prep Kit (Cat. # KK8500, KK4824 and KK8502). Libraries were subjected to 100 bp paired-end sequencing on an Illumina NovaSeq 6000.

Hi-C sequencing data analysis

Adapters and low-quality reads were removed using bbmap and bbduk.sh (https://jgi.doe.gov/data-and-tools/bbtools/bb-tools-user-guide/bbduk-guide/). Reads were trimmed using Homer (version 4.10) [18]. PCR duplicates were removed using Picard MarkDuplicates (version 2.0.1), and underwent quality control by FastQC program [14]. Filtered reads were mapped to the mouse (mm9) or human (hg19) reference genome using Bowtie2 with parameters ("-n 1 -m 1 -p 8") [5]. Mapped sequencing data was used to generate a contact matrix using Homer (version 4.10). Then, analyzeHiC program was performed to generate a normalized

and visualizable interaction matrix with default parameters, and the intra-chromatin interactions of the specific loci were generated by analyzeHiC program in Homer/4.10 with parameters (-res 10,000 -superRes 20,000 -pos chromosome location). ANOVA analysis was performed to identify TADs with significant variability in domain score by comparison of WT and XPO1 treated AML cells, or WT and *NPM1c+* murine cells (cutoff: Bonferroni-corrected p value < 0.05). The differential chromatin interactions were evaluated through HiCExplorer (version 3.5.3) [22]. These normalized and visualized chromatin interaction matrices were used to generate the Hi-C heatmap using juicer (version 1.5.5) [23], and visualized with Juicebox [24]. Furthermore, the domain score of the TAD was normalized by subtracting the mean of all TADs, and quantile-normalization was applied on domain scores to facilitate comparison among all Hi-C signals with HOMER program (v4.10) [25]. All genomics datasets were deposited in the NCBI GEO under accession number (GEO: GSE208022).

Xenotransplantation of human leukemic cells

Adult NOD.*Cg-Prkdc*^{scid}*Il2rg*^{tm1Wjl}/SzJ (NSG) mice (6-8 weeks old) were blindly transplanted each test group with $5x10^5$ OCI-AML3 by tail-vein injection. At 35 days after transplantation, peripheral blood (PB) was collected and depleted of red blood cells by ammonium chloride treatment, BM was isolated from the tibias, femurs, and pelvis. Human $CD45^+$ chimerism in the BM and PB cells were analyzed with anti-human CD45 (BD, CD45 Mouse Anti- Human, APC, Clone: HI30) by flow cytometry analysis of hCD45 chimerism in PB (FACS LSR II–BD Biosciences, San Jose, CA, USA). All animal studies were conducted in accordance with the regulatory guidelines by the Institutional Animal Care and Use Committee (IACUC) at Penn State Hershey Medical Center.

Quantification and statistical analysis

Statistical differences were determined by Student's t-test (after testing for normal distribution) or analysis of variance (ANOVA) followed by Newman-Keuls multiple comparison

tests. For *in vitro* experiments, at least three independent experiments with at least three biological replicates for each condition/genotype were carried out. For *in vivo* experiments, the sample size of five mice/group/genotype containing both male and female animals at similar age (6-10 weeks old) were chosen and animals were randomly assigned to each study arm.

References:

1 Mallardo, M., Caronno, A., Pruneri, G., Raviele, P.R., Viale, A., Pelicci, P.G., *et al.* NPMc+ and FLT3_ITD mutations cooperate in inducing acute leukaemia in a novel mouse model. *Leukemia* **27**, 2248-2251, doi:10.1038/leu.2013.114 (2013).

2 Brunetti, L., Gundry, M.C., Sorcini, D., Guzman, A.G., Huang, Y.H., Ramabadran, R., *et al.* Mutant NPM1 Maintains the Leukemic State through HOX Expression. *Cancer Cell* **34**, 499-512.e499, doi:10.1016/j.ccell.2018.08.005 (2018).

3 Martin, M. Cutadapt Removes Adapter Sequences from High-Throughput Sequencing Reads. *EMBnet Journal* **17**, 10-12 (2011).

4 Trapnell, C., Roberts, A., Goff, L., Pertea, G., Kim, D., Kelley, D.R., *et al.* Differential gene and transcript expression analysis of RNA-seq experiments with TopHat and Cufflinks. *Nat Protoc* **7**, 562-578, doi:10.1038/nprot.2012.016 (2012).

5 Langmead, B., Trapnell, C., Pop, M. & Salzberg, S. L. Ultrafast and memory-efficient alignment of short DNA sequences to the human genome. *Genome Biol* **10**, R25, doi:10.1186/gb-2009-10-3-r25 (2009).

6 Trapnell, C., Pachter, L. & Salzberg, S. L. TopHat: discovering splice junctions with RNA-Seq. *Bioinformatics* **25**, 1105-1111, doi:10.1093/bioinformatics/btp120 (2009).

7 Trapnell, C., Williams, B.A., Pertea, G., Mortazavi, A., Kwan, G., Baren, M.J.V., *et al.* Transcript assembly and quantification by RNA-Seq reveals unannotated transcripts and isoform switching during cell differentiation. *Nat Biotechnol* **28**, 511-515, doi:10.1038/nbt.1621 (2010).

8 de Hoon, M. J., Imoto, S., Nolan, J. & Miyano, S. Open source clustering software. *Bioinformatics* **20**, 1453-1454, doi:10.1093/bioinformatics/bth078 (2004).

9 Subramanian, A., Tamayo, P., Mootha, V.K., Mukherjee, S., Ebert, B.L., Gillette, M.A., *et al.* Gene set enrichment analysis: a knowledge-based approach for interpreting genome-wide expression profiles. *Proc Natl Acad Sci U S A* **102**, 15545-15550, doi:10.1073/pnas.0506580102 (2005).

10 Huang da, W., Sherman, B. T. & Lempicki, R. A. Systematic and integrative analysis of large gene lists using DAVID bioinformatics resources. *Nat Protoc* **4**, 44-57, doi:10.1038/nprot.2008.211 (2009).

11 Luo, H., Wang, F., Zha, J., Li, H., Yan, B., Du, Q., *et al.* CTCF boundary remodels chromatin domain and drives aberrant HOX gene transcription in acute myeloid leukemia. *Blood* **132**, 837-848, doi:10.1182/blood-2017-11-814319 (2018).

12 Buenrostro, J. D., Wu, B., Chang, H. Y. & Greenleaf, W. J. ATAC-seq: A Method for Assaying Chromatin Accessibility Genome-Wide. *Curr Protoc Mol Biol* **109**, 21 29 21-29, doi:10.1002/0471142727.mb2129s109 (2015).

13 Corces, M.R., Trevino, A.E., Hamilton, E.G., Greenside, P.G., Sinnott-Armstrong, N.A., Vesuna, S., *et al.* An improved ATAC-seq protocol reduces background and enables

interrogation of frozen tissues. *Nat Methods* **14**, 959-962, doi:10.1038/nmeth.4396 (2017).
14 Wingett, S. W. & Andrews, S. FastQ Screen: A tool for multi-genome mapping and quality control. *F1000Res* **7**, 1338, doi:10.12688/f1000research.15931.2 (2018).
15 Li, H., Handsaker, B., Wysoker, A., Fennell, T., Ruan, J., Homer, N., *et al.* The Sequence Alignment/Map format and SAMtools. *Bioinformatics* **25**, 2078-2079, doi:10.1093/bioinformatics/btp352 (2009).
16 Zhang, Y., Liu, T., Meyer, C.A., Eeckhoute, J., Johnson, D.S., Bernstein, B.E., *et al.* Model-based analysis of ChIP-Seq (MACS). *Genome Biol* **9**, R137, doi:10.1186/gb-2008-9-9-r137 (2008).
17 Robinson, J.T., Thorvaldsdóttir, H., Winckler, W., Guttman, M., Lander, E.S., Getz, G., *et al.* Integrative genomics viewer. *Nat Biotechnol* **29**, 24-26, doi:10.1038/nbt.1754 (2011).
18 Heinz, S., Benner, C., Spann, N., Bertolino, E., Lin, Y.C., Laslo, P., *et al.* Simple combinations of lineage-determining transcription factors prime cis-regulatory elements required for macrophage and B cell identities. *Mol Cell* **38**, 576-589, doi:10.1016/j.molcel.2010.05.004 (2010).
19 Ross-Innes, C.S., Stark, R., Teschendorff, A.E., Holmes, K.A., Ali, H.R., Dunning, M.J., *et al.* Differential oestrogen receptor binding is associated with clinical outcome in breast cancer. *Nature* **481**, 389, doi:10.1038/nature10730

https://www.nature.com/articles/nature10730#supplementary-information (2012).
20 Patel, B., Kang, Y., Cui, K., Litt, M., Riberio, M.S.J., Deng, C., *et al.* Aberrant TAL1 activation is mediated by an interchromosomal interaction in human T-cell acute lymphoblastic leukemia. *Leukemia* **28**, 349-361, doi:10.1038/leu.2013.158 (2014).
21 Deng, C., Li, Y., Zhou, L., Cho, J., Patel, B., Terada, N., *et al.* HoxBlinc RNA Recruits Set1/MLL Complexes to Activate Hox Gene Expression Patterns and Mesoderm Lineage Development. *Cell Rep* **14**, 103-114, doi:10.1016/j.celrep.2015.12.007 (2016).
22 Wolff, J., Rabbani, L., Gilsbach, R., Richard, G., Manke, T., Backofen, R., *et al.* Galaxy HiCExplorer 3: a web server for reproducible Hi-C, capture Hi-C and single-cell Hi-C data analysis, quality control and visualization. *Nucleic Acids Res* **48**, W177-W184, doi:10.1093/nar/gkaa220 (2020).
23 Durand, N.C., Shamim, M.S., Machol, I., Rao, S.S.P., Huntley, M.H., Lander, E.S., *et al.* Juicer Provides a One-Click System for Analyzing Loop-Resolution Hi-C Experiments. *Cell Syst* **3**, 95-98, doi:10.1016/j.cels.2016.07.002 (2016).
24 Durand, N.C., Robinson, J.T., Shamim, M.S., Machol, I., Mesirov, J.P., Lander, E.S., *et al.* Juicebox Provides a Visualization System for Hi-C Contact Maps with Unlimited Zoom. *Cell Syst* **3**, 99-101, doi:10.1016/j.cels.2015.07.012 (2016).
25 Lin, Y.C., Benner, C., Mansson, R., Heinz, S., Miyazaki, K., Miyazaki, M., *et al.* Global changes in the nuclear positioning of genes and intra- and interdomain genomic interactions that orchestrate B cell fate. *Nat Immunol* **13**, 1196-1204, doi:10.1038/ni.2432 (2012).

Supplementary Figures and legends:

Supplementary Figure 1. *NPM1^{C+}* KI in mouse BM HS/PCs reprograms Hoxa and Hoxb TAD topology and transcription signatures. (A) Volcano plot analysis of RNA-seq shows that

posterior *Hoxa*, anterior *Hoxb*, and myeloid master TF *Runx1* genes were activated upon *NPM1^{C+}* KI for 4 months. **(B)** ATAC-seq analysis of chromatin accessibility of Hoxa (Top) and Hoxb (Bottom) loci comparing WT and *NPM1^{C+}* KI mouse BM HS/PCs upon induction of NPM1^{C+} for 4 months.

Supplementary Figure 2. Nuclear retention of NPM1 enhanced NPM1 and CTCF interaction and alters leukemic TAD formation and gene expression. (A) Overlapping upregulated (Top) or downregulated (Bottom) genes by RNA-seq between XPO1i treatment and *NPM1^{OE}* OCI-AML3 cells. **(B)** GO term analysis of overlapped upregulated or downregulated gene by XPO1i treatment and *NPM1^{OE}*. **(C)** GO term analysis of overlapping of genes encompassed in the enhanced (Left) or decreased (Right) TADs identified by Hi-C assay and upregulated (Left) or downregulated (Right) genes determined by RNA-seq in the WT *NPM1^{OE}* OCI-AML3 cells (Left), respectively. **(D)** Overlapping of enhanced or reduced TAD encompassed genes and downregulated (Left) or upregulated (Right) genes by RNA-seq in the WT *NPM1^{OE}* OCI-AML3 cells, respectively. **(E)** Overlapping of genes encompassed in the enhanced TADs identified by Hi-C assay and upregulated genes determined by RNA-seq in the XPO1i treated OCI-AML3 cells (Left). GO term analysis of overlapped enhanced TAD encompassed genes and upregulated genes upon XPO1i treated OCI-AML3 cells (Right). **(F)** Overlapping of genes encompassed in the reduced TADs identified by Hi-C assay and downregulated genes determined by RNA-seq in the XPO1i treated OCI-AML3 cells (Left). GO term analysis of overlapped reduced TAD encompassed genes and downregulated genes upon XPO1i treated OCI-AML3 cells (Right).

Supplementary Figure 3. Nuclear relocation of NPM1^{C+} alters CTCF-driven loop structure required for NPM1^{C+} transcription. (A) WB analysis of cytoplasmic and nuclear NPM1 protein levels of OCI-AML3 cells upon time course treatment of 50 nM Selinexor. **(B)** immunofluorescent staining of cytoplasmic and nuclear localization of NPM1 protein in OCI-

AML3 cells upon time course treatment of 50 nM Selinexor. **(C)** Quantification of NPM1 positive nuclei upon time course Selinexor treatment of OCI-AML3 cells (Right). **(D)** Chromatin conformation Capture (3C) analysis of CTCF mediated chromatin loops in the *HOXB* and *CEBPA/G* loci upon 0hr, 3hr, or 24hr Selinexor treatment. Left: schematic representation of chromatin loops detected in the *HOXB* (Top) and *CEBPA* (Bottom) loci by Hi-C. Right: quantitation of 3C bands from two technical repeats. **(E)** RT-qPCR analysis of a few NPM1 signature gene expression in OCI-AML3 cells treated with 50 nM Selinexor for 0 or 24 hr. Data are presented as mean ± SD. $^{*}p \leq 0.05$; $^{**}p \leq 0.01$; $^{***}p \leq 0.001$.

Supplementary Figure 4. Nuclear retention of NPM1 switched MIZ-1 from transcription repressor to transcription activator by altering its interaction with corepressor MYC/G9A to coactivator NPM1/p300. (A) co-immunoprecipitation of MIZ-1 interacting proteins comparing WT and XPO1i treated or $NPM1^{OE}$ OCI-AML3 nuclear extracts. **(B)** RT-qPCR examination of expression levels of MIZ-1 target genes comparing WT and XPO1i treated or $NPM1^{OE}$ OCI-AML3 cells. **(C)** ChIP-qPCR analysis of MIZ-1 chromatin binding at MIZ-1 target genes comparing WT and XPO1i treated or $NPM1^{OE}$ OCI-AML3 cells. **(D)** ChIP-qPCR analysis of c-MYC chromatin binding at MIZ-1 target genes comparing WT and XPO1i treated or $NPM1^{OE}$ OCI-AML3 cells. **(E)** ChIP-qPCR analysis of G9A chromatin binding at MIZ-1 target genes comparing WT and XPO1i treated or $NPM1^{OE}$ OCI-AML3 cells. **(F)** ChIP-qPCR analysis of NPM1 chromatin binding at MIZ-1 target genes comparing WT and XPO1i treated or $NPM1^{OE}$ OCI-AML3 cells. **(G)** ChIP-qPCR analysis of EP300 chromatin binding at MIZ-1 target genes comparing WT and XPO1i treated or $NPM1^{OE}$ OCI-AML3 cells. **(H)** ChIP-qPCR analysis of H3K27ac enrichment at MIZ-1 target genes comparing WT and XPO1i treated or $NPM1^{OE}$ OCI-AML3 cells. **(I)** ChIP-qPCR analysis of H3K9me2 enrichment at MIZ-1 target genes comparing WT and XPO1i treated or $NPM1^{OE}$ OCI-AML3 cells. **(J)** ChIP-qPCR analysis of IgG control in the target loci. Data in (B)-(J) are presented as mean ± SD. $^{*}p \leq 0.05$; $^{**}p \leq 0.01$; $^{***}p \leq 0.001$.

(K) Publicly available Bru-seq datasets from OCI-AML3 cells treated with or without XPO1i, Selinexor, (GSE197387) were reanalyzed for nascent transcripts enrichment in *CDKN1A* and *CEBPA* loci.

Supplementary Figure 5. Nuclear retention of NPM1 switched MYC/MIZ-1 axis to block aberrant cell cycle and promote myeloid differentiation. (A) May-Giemsa staining of OCI-AML3 cells comparing WT, XPO1i treated, or $NPM1^{OE}$ OCI-AML3 cells. **(B)** FACS analysis of CD11b and CD14 double positive cells comparing WT, XPO1i treated, or $NPM1^{OE}$ OCI-AML3 cells. **(C)** Quantitation of CD11b and CD14 double positive cells comparing WT, XPO1i treated, or $NPM1^{OE}$ OCI-AML3 cells. **(D)** FACS analysis of the cell cycle was performed using propidium iodide staining of WT, XPO1i treated, or $NPM1^{OE}$ OCI-AML3 cells. **(E)** FACS analysis of Annexin V positive apoptotic cells in WT, XPO1i treated, or $NPM1^{OE}$ OCI-AML3 cells. **(F)** Ki67 antibody staining of proliferative cells comparing WT, XPO1i treated, or $NPM1^{OE}$ OCI-AML3 cells.

A

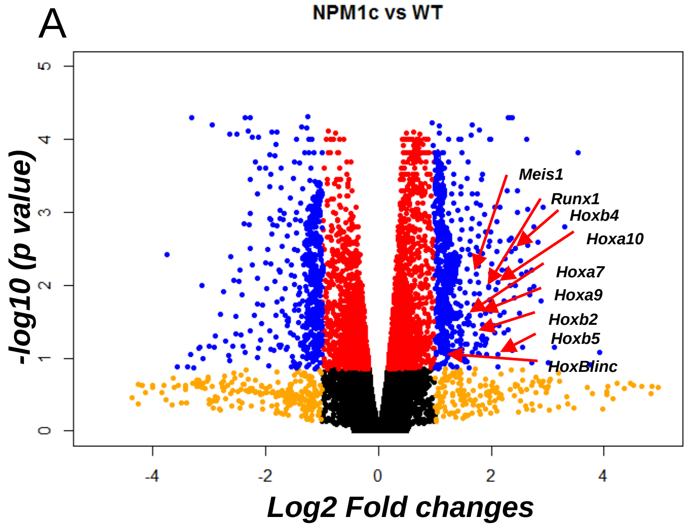
NPM1c vs WT
-log10 (p value)
Log2 Fold changes
Meis1
Runx1
Hoxb4
Hoxa10
Hoxa7
Hoxa9
Hoxb2
Hoxb5
HoxBlinc

B

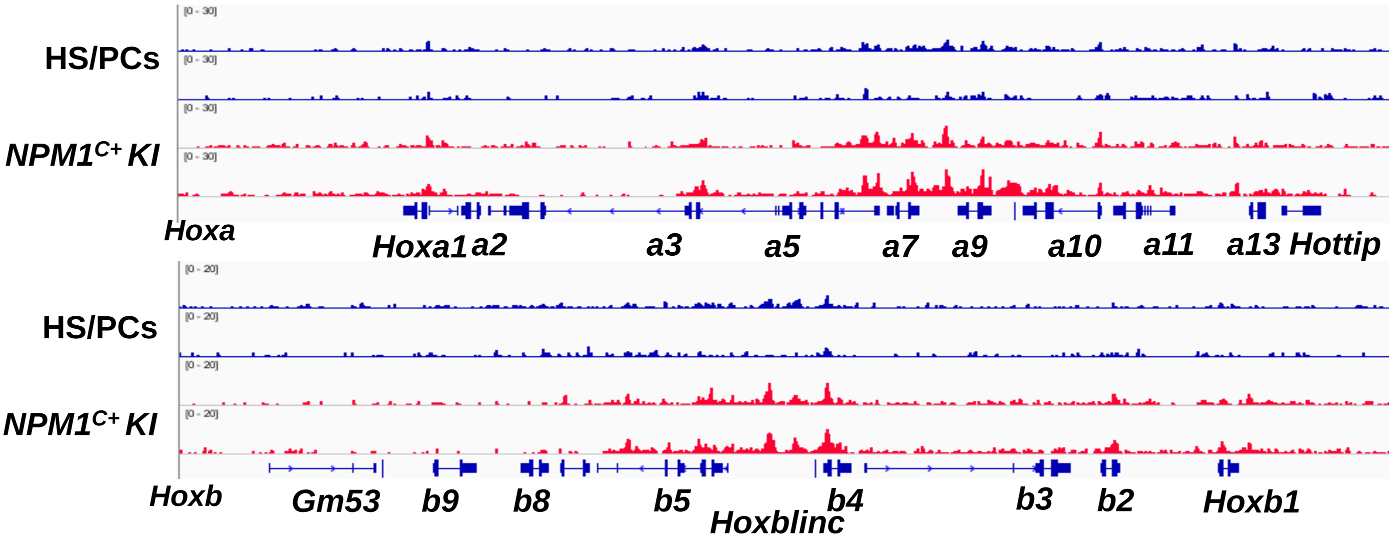
HS/PCs
NPM1C+ KI
Hoxa
Hoxa1 a2
a3
a5
a7
a9
a10
a11
a13 Hottip
HS/PCs
NPM1C+ KI
Hoxb
Gm53
b9
b8
b5
Hoxblinc
b4
b3
b2
Hoxb1

Figure S2

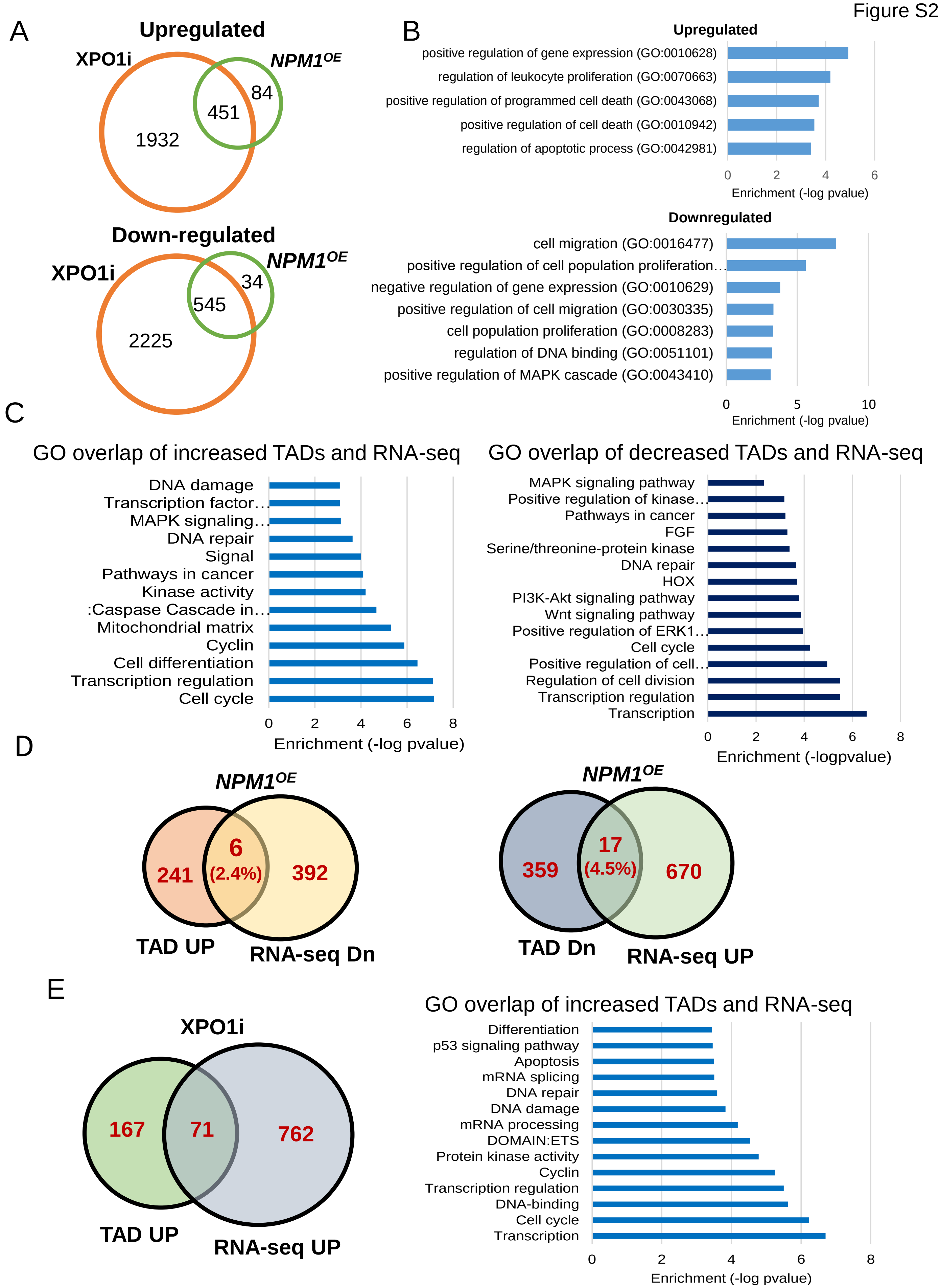

A
Upregulated
XPO1i
NPM1OE
1932
451
84
Down-regulated
XPO1i
NPM1OE
2225
545
34
B
Upregulated
positive regulation of gene expression (GO:0010628)
regulation of leukocyte proliferation (GO:0070663)
positive regulation of programmed cell death (GO:0043068)
positive regulation of cell death (GO:0010942)
regulation of apoptotic process (GO:0042981)
0 2 4 6
Enrichment (-log pvalue)
Downregulated
cell migration (GO:0016477)
positive regulation of cell population proliferation…
negative regulation of gene expression (GO:0010629)
positive regulation of cell migration (GO:0030335)
cell population proliferation (GO:0008283)
regulation of DNA binding (GO:0051101)
positive regulation of MAPK cascade (GO:0043410)
0 5 10
Enrichment (-log pvalue)
C
GO overlap of increased TADs and RNA-seq
DNA damage
Transcription factor…
MAPK signaling…
DNA repair
Signal
Pathways in cancer
Kinase activity
:Caspase Cascade in…
Mitochondrial matrix
Cyclin
Cell differentiation
Transcription regulation
Cell cycle
0 2 4 6 8
Enrichment (-log pvalue)
GO overlap of decreased TADs and RNA-seq
MAPK signaling pathway
Positive regulation of kinase…
Pathways in cancer
FGF
Serine/threonine-protein kinase
DNA repair
HOX
PI3K-Akt signaling pathway
Wnt signaling pathway
Positive regulation of ERK1…
Cell cycle
Positive regulation of cell…
Regulation of cell division
Transcription regulation
Transcription
0 2 4 6 8
Enrichment (-logpvalue)
D
NPM1OE
241
6
(2.4%)
392
TAD UP
RNA-seq Dn
NPM1OE
359
17
(4.5%)
670
TAD Dn
RNA-seq UP
E
XPO1i
167
71
762
TAD UP
RNA-seq UP
GO overlap of increased TADs and RNA-seq
Differentiation
p53 signaling pathway
Apoptosis
mRNA splicing
DNA repair
DNA damage
mRNA processing
DOMAIN:ETS
Protein kinase activity
Cyclin
Transcription regulation
DNA-binding
Cell cycle
Transcription
0 2 4 6 8
Enrichment (-log pvalue)

F

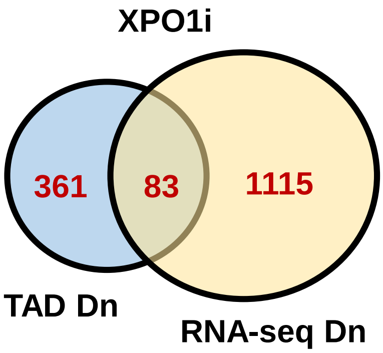
XPO1i
361
83
1115
TAD Dn
RNA-seq Dn

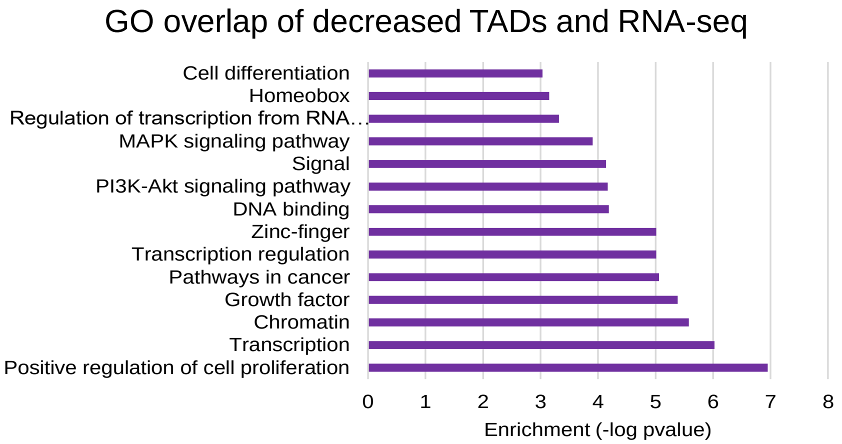
GO overlap of decreased TADs and RNA-seq
Cell differentiation
Homeobox
Regulation of transcription from RNA...
MAPK signaling pathway
Signal
PI3K-Akt signaling pathway
DNA binding
Zinc-finger
Transcription regulation
Pathways in cancer
Growth factor
Chromatin
Transcription
Positive regulation of cell proliferation
0
1
2
3
4
5
6
7
8
Enrichment (-log pvalue)

Figure S3

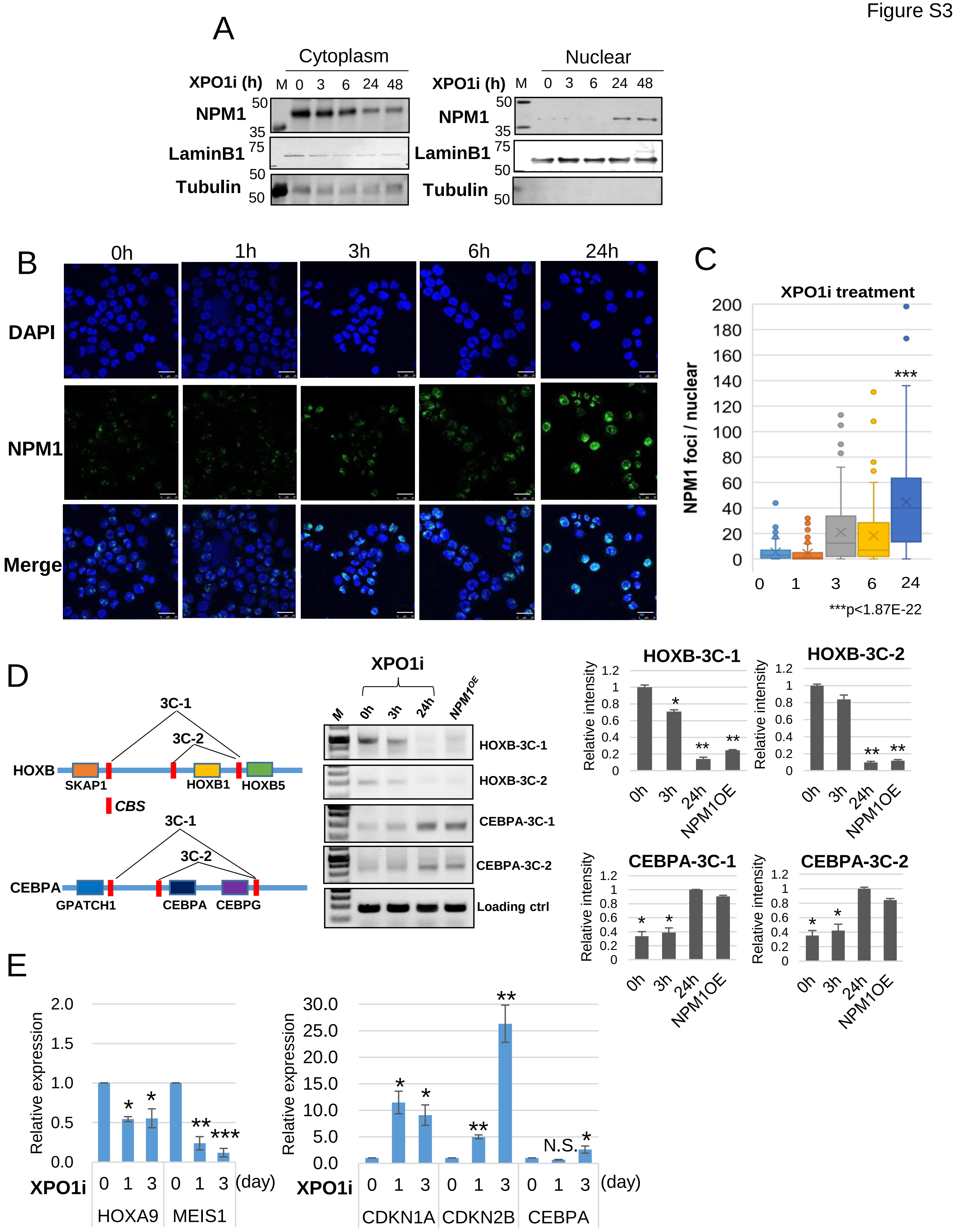

A
Cytoplasm
Nuclear
XPO1i (h)
M 0 3 6 24 48
NPM1
LaminB1
Tubulin
50
35
75
50
B
0h
1h
3h
6h
24h
DAPI
NPM1
Merge
C
XPO1i treatment
NPM1 foci / nuclear
***
***p<1.87E-22
D
HOXB
SKAP1
HOXB1
HOXB5
3C-1
3C-2
CBS
CEBPA
GPATCH1
CEBPG
XPO1i
M
0h
3h
24h
NPM1OE
HOXB-3C-1
HOXB-3C-2
CEBPA-3C-1
CEBPA-3C-2
Loading ctrl
Relative intensity
E
Relative expression
XPO1i
(day)
HOXA9
MEIS1
CDKN1A
CDKN2B
CEBPA
N.S.

Figure S4

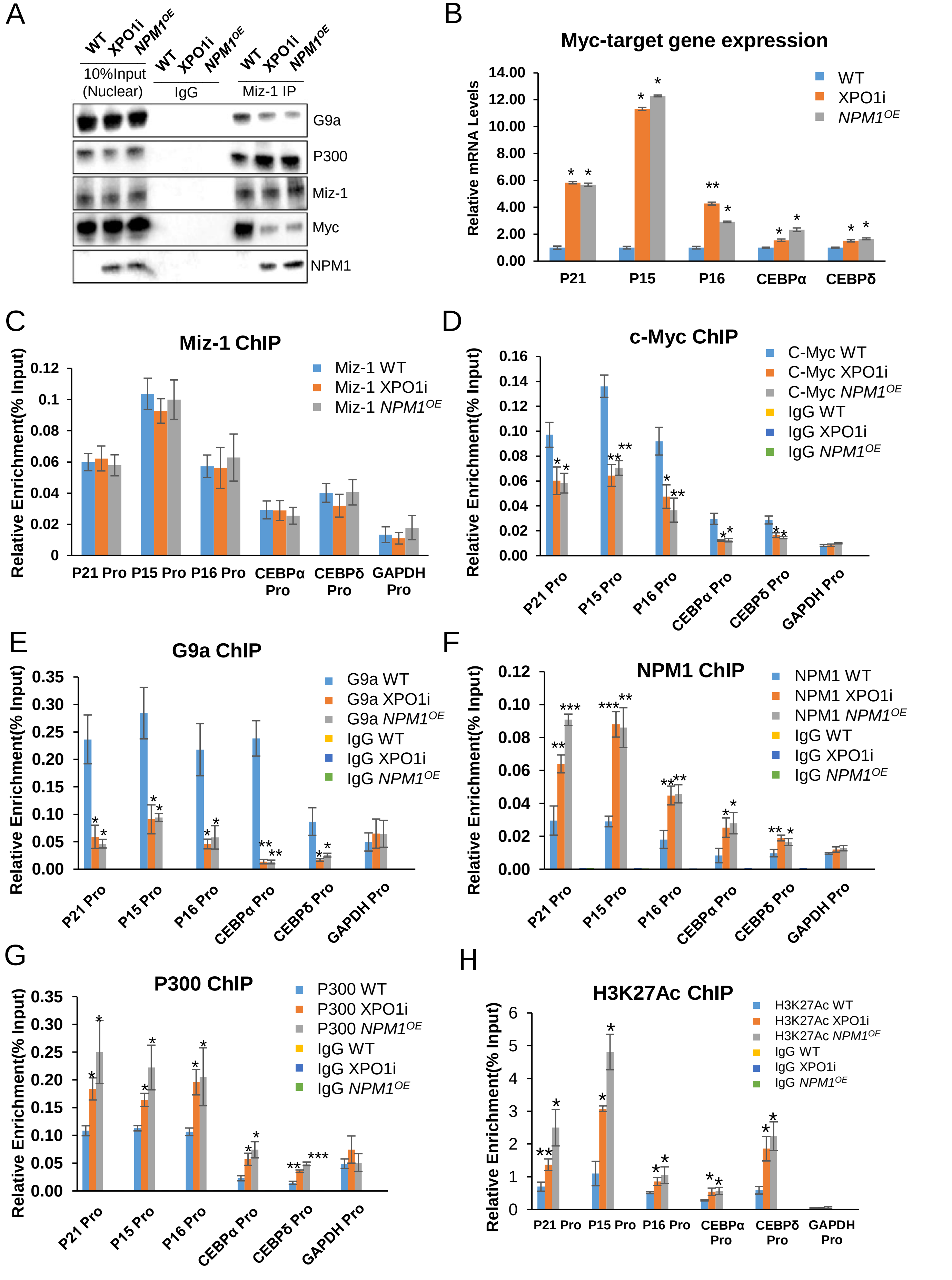
A
WT XPO1i NPM1OE
10%Input (Nuclear)
IgG
Miz-1 IP
G9a
P300
Miz-1
Myc
NPM1
B
Myc-target gene expression
Relative mRNA Levels
WT
XPO1i
NPM1OE
P21
P15
P16
CEBPα
CEBPδ
C
Miz-1 ChIP
Relative Enrichment(% Input)
Miz-1 WT
Miz-1 XPO1i
Miz-1 NPM1OE
P21 Pro
P15 Pro
P16 Pro
CEBPα Pro
CEBPδ Pro
GAPDH Pro
D
c-Myc ChIP
C-Myc WT
C-Myc XPO1i
C-Myc NPM1OE
IgG WT
IgG XPO1i
IgG NPM1OE
E
G9a ChIP
G9a WT
G9a XPO1i
G9a NPM1OE
F
NPM1 ChIP
NPM1 WT
NPM1 XPO1i
NPM1 NPM1OE
G
P300 ChIP
P300 WT
P300 XPO1i
P300 NPM1OE
H
H3K27Ac ChIP
H3K27Ac WT
H3K27Ac XPO1i
H3K27Ac NPM1OE

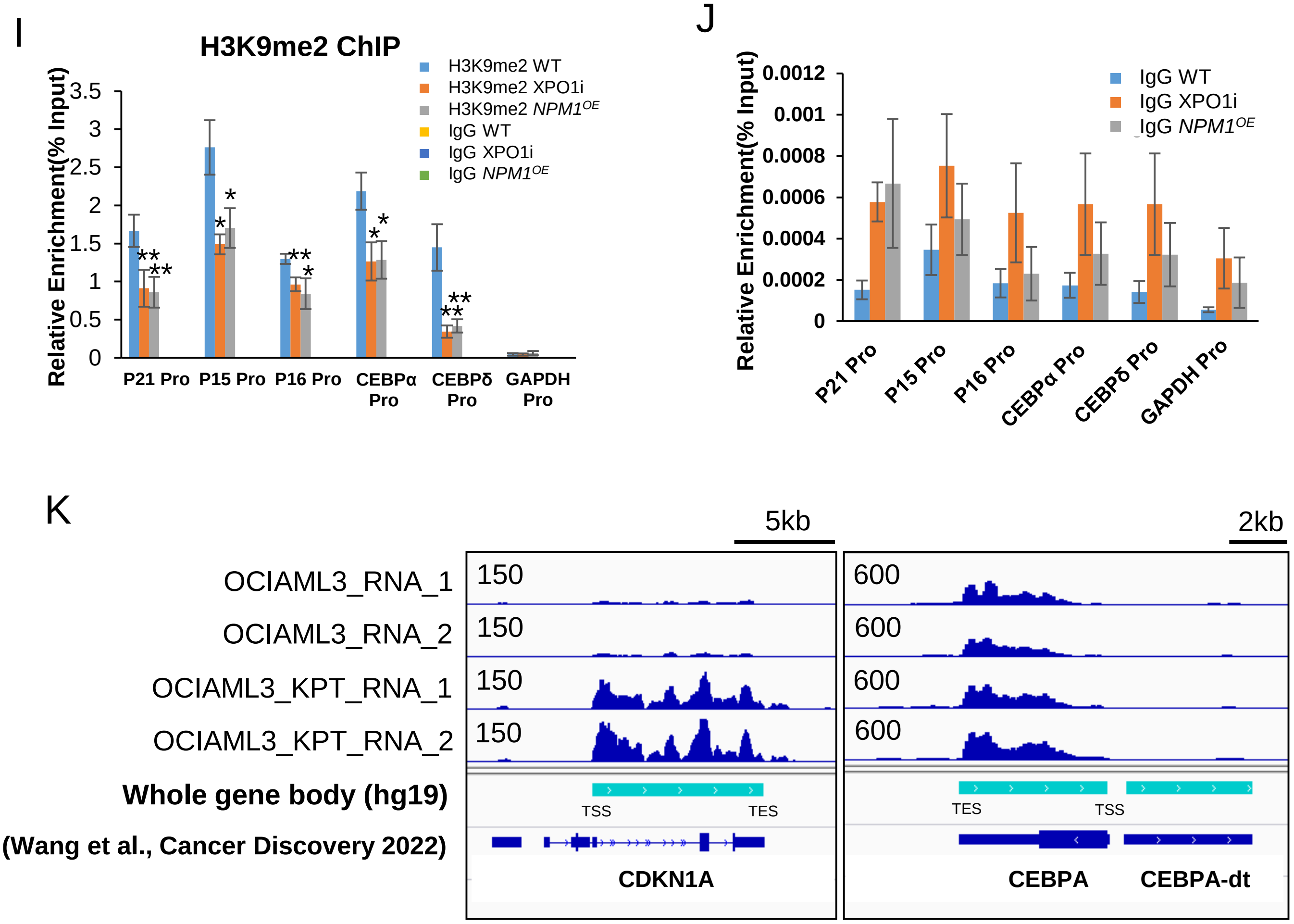
I
H3K9me2 ChIP
H3K9me2 WT
H3K9me2 XPO1i
H3K9me2 NPM1OE
IgG WT
IgG XPO1i
IgG NPM1OE
Relative Enrichment(% Input)
3.5
3
2.5
2
1.5
1
0.5
0
P21 Pro
P15 Pro
P16 Pro
CEBPα Pro
CEBPδ Pro
GAPDH Pro
J
IgG WT
IgG XPO1i
IgG NPM1OE
Relative Enrichment(% Input)
0.0012
0.001
0.0008
0.0006
0.0004
0.0002
0
P21 Pro
P15 Pro
P16 Pro
CEBPα Pro
CEBPδ Pro
GAPDH Pro
K
5kb
2kb
OCIAML3_RNA_1
OCIAML3_RNA_2
OCIAML3_KPT_RNA_1
OCIAML3_KPT_RNA_2
150
600
Whole gene body (hg19)
(Wang et al., Cancer Discovery 2022)
TSS
TES
CDKN1A
CEBPA
CEBPA-dt

Figure S5

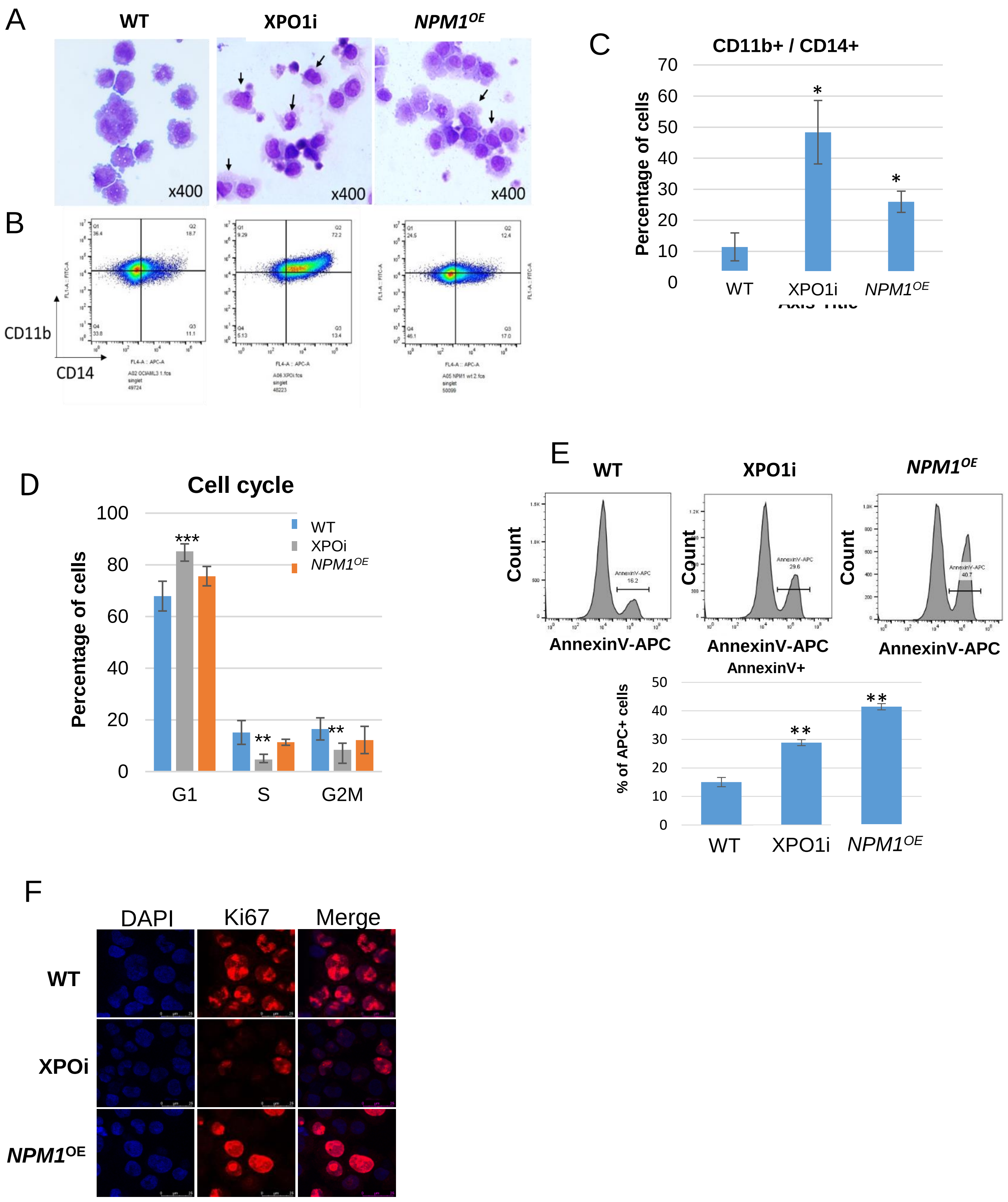
A
WT
XPO1i
NPM1OE
x400
x400
x400
B
CD11b
CD14
C
CD11b+ / CD14+
Percentage of cells
70
60
50
40
30
20
10
0
*
*
WT
XPO1i
NPM1OE
D
Cell cycle
Percentage of cells
100
80
60
40
20
0
***
**
**
WT
XPOi
NPM1OE
G1
S
G2M
E
WT
XPO1i
NPM1OE
Count
Count
Count
AnnexinV-APC
AnnexinV-APC
AnnexinV-APC
AnnexinV+
% of APC+ cells
50
40
30
20
10
0
**
**
WT
XPO1i
NPM1OE
F
DAPI
Ki67
Merge
WT
XPOi
NPM1OE
